# During the Long Way to Mars: Effects of 520 Days of Confinement (Mars500) on the Assessment of Affective Stimuli and Stage Alteration in Mood and Plasma Hormone Levels

**DOI:** 10.1371/journal.pone.0087087

**Published:** 2014-04-02

**Authors:** Yue Wang, Xiaolu Jing, Ke Lv, Bin Wu, Yanqiang Bai, Yuejia Luo, Shanguang Chen, Yinghui Li

**Affiliations:** 1 National Key Laboratory of Cognitive Neuroscience and learning, Beijing Normal University, Beijing, China; 2 State Key Laboratory of Space Medicine Fundamentals and Application, China Astronaut Research and Training Center, Beijing, China; 3 China Astronaut Research and Training Center, Beijing, China; 4 Institute of Affective and Social Neuroscience, Shenzhen University, Shenzhen, China; 5 National Laboratory of Human Factors Engineering, China Astronaut Research and Training Center, Beijing, China; National Space Biomedical Research Institute, United States of America

## Abstract

For future interplanetary manned spaceflight, mental issues, as well as physiological problems, must inevitably be considered and solved. Mars500 is a high-fidelity ground simulation experiment that involved 520 days of confined isolation for six multinational crewmembers. This experiment provided a good opportunity to perform psycho-physiological and psycho-social researches on such missions. To investigate emotional responses and psychological adaptation over long-term confinement, the International Affective Pictures System (IAPS) was selected as the visual emotional stimuli in this study. Additional data collected and analyzed included the Profile of Mood States (POMS) questionnaire and the levels of four types of plasma hormones: cortisol, 5-hydroxy tryptamine, dopamine, and norepinephrine. The results demonstrated an obvious bias on valence rating for unpleasant stimuli with time (*p*<0.05), and the correlation between psychological and biochemical data was identified (*p*<0.05). Overall, we concluded that the confined crew tended to assign positive ratings to negative pictures with time, which might be driven by a defensive system. There was a stage-changing pattern of psychological adaptation of the Mars500 crew, which is similar to the third-quarter phenomenon.

## Introduction

The identification of psychological problems and their appropriate countermeasures must be taken into consideration for future long-term interplanetary manned space missions [1], [2], [3], [4]. Aside from actual spaceflights, a number of similar expeditions under isolated and confined environments (ICEs) have been performed to study human mental issues, including Antarctic exploration [5], [6], [7], submarine mission [8], ground-based spaceflight simulation, and others [9], [10], [11], [12]. Previous studies have found evidence of decrement in the variables of cognitive performance [13], [14], [15], team cohesion [1], [3], [4], [16], locomotor function [10], [11], [17], circadian rhythm and sleep [2], [3], [18], [19], etc. Moreover, the strong association between the function of the neuroendocrine system and emotion processing and stress coping has also been observed [20], [21], [22], [23]. To the best of our knowledge, however, the effect of long-term confinement on the assessment of affective stimuli has not yet been reported.

A cluster of symptoms has been described as the winter-over syndrome, consisting of sleep disturbance, impaired cognition, negative affect, and interpersonal tension and conflict experienced by people on polar expeditions in the Antarctic [5]. Several studies have noted that these symptoms seem to increase after the midpoint of an expedition and are reduced toward the end of the expedition [6], [7], [15]. This pattern is known as the third-quarter phenomenon, and it seems to be due to the psychosocial factors rather than the environment. It refers to the psycho-physiological changes over a period of time in space as well as in Antarctica, particularly with the presence of increased mental issues during the third quarter in the company. This phenomenon most likely results from the realization that the mission is only half completed, and a long period of isolation still awaits [1], [4], [5], [16]. However, some empirical findings have argued against the existence of this phenomenon [7], [11], [14], [16]. Due to the special and strict experiment requirements, the progress of such research has been limited.

The Mars500 project, which simulated an interplanetary trip between the Earth and Mars, provided an outstanding opportunity to research the changes in psychological adaptation over 520 days of confinement and isolation. To investigate the emotional response over time, we selected affective pictures from International Affective Picture System (IAPS) as visual affective stimuli. The subjective questionnaire called Profiles of Mood State (POMS) was also performed to acquire data about the crew's mental state. Furthermore, we collected and analyzed four types of plasma hormones: cortisol, 5-hydroxy tryptamine (5-HT), dopamine (DA) and norepinephrine (NE) over the course of the confinement. Intriguingly, this study identified a positive rating bias toward unpleasant stimuli and a stage-changing pattern in the psychological adaptation of the Mars500 crew.

## Materials and Methods

### Mars500 mission

The Mars500 project was implemented to simulate a flight to Mars to prepare for future deep-space exploration. It lasted 520 days, from Jun 3^rd^ 2010 to Nov 4^th^ 2011. Six male participants (three Russians, two Europeans, and one Chinese, aged 32.4±4.8 years) were selected worldwide as the Mars500 crew with the consideration of psycho-physiological health, language, occupation background and so on. The experimental facility was located in the Institute of Biomedical Problems (IBMP) of the Russian Academy of Science (RSA), Moscow. The Mars500 crew lived in this spaceship-like habitat with continuous temporal and spatial isolation, realistic mission activities, a diurnal weekly work schedule, communication lag, a mid-mission landing on a simulated Mars surface and other major special conditions of a Martian flight. Detailed information about all crewmembers and the entire project is available on the internet (http://mars500.imbp.ru/en/index_e.html).

This research was conducted in accordance with the principles expressed in the Declaration of Helsinki. The ethical committee of the Institute of Biomedical Problems approved this study, and all Mars500 crewmembers gave their written informed consent.

### Tests

#### International Affective Pictures System (IAPS)

Researchers use many diverse methods to investigate emotion. The International Affective Pictures System (IAPS) is a specific image set containing various pictures depicting snakes, insects, accidents, illness, puppies, babies, and landscape scenes, among others. Each picture must be rated on two dimensions, valence and arousal. Valence means the intrinsic attractiveness (positive) or aversiveness (negative) of stimuli, and arousal means a psychophysiological state of being awake (low) or reactive (high) to stimuli. In IAPS,the valence rating ranges from unpleasant (low pleasure) to pleasant (high pleasure), and arousal rating ranges from calm (low arousal) to excited (high arousal). The collections of norms for affective pictures are useful for selecting suitable affective stimuli and for making comparisons [24], [25]. With regard to the safety and maneuverability, we chose IAPS to study affective assessment in the present study.

In total, there were 295 different affective pictures selected from the IAPS, depicting 61 pleasant events (i.e., attractive infants, family), 61 unpleasant events (i.e., snakes, bleeding, catastrophe) and 173 neutral events (i.e., neutral face, household objects), for which the levels of pleasantness differed significantly depending on the normative valence. Based on the method of exposure, these pictures were divided into two groups: Repetitive Group (RG) and Novel Group (NG). In this study, there were 8 sessions of image tests in all, each of which contained one RG and one NG. The RG comprised 15 pictures of equal size and composition (5 pleasant, 5 unpleasant and 5 neutral stimuli) that were presented repeatedly every time. For the NG, 35 different, novel pictures (7 pleasant, 7 unpleasant and 21 neutral) were randomly arranged in each session and mixed with pictures repeated from the RG. Therefore, each crewmember was required to evaluate 50 affective images on two dimensions. Both valence and arousal were rated on a nine-point scale (valence: ‘1’ = lowest pleasure, ‘9’ = highest pleasure; arousal: ‘1’ = lowest arousal, ‘9’ = high arousal). There was no difference between the normative valences of RG and NG. The normative arousal of pleasant pictures in RG was lower than that in NG (table 1).

**Table 1:** Normative values of the selected affective pictures from IAPS.

	Valence						Arousal					
	pleasant		neutral		unpleasant		low		neutral		high	
	mean	SD	mean	SD	mean	SD	mean	SD	mean	SD	mean	SD
Repetitive Group (n = 15)	7.375	0.421	5.298	0.977	2.78	0.528	4.063	1.042	4.853	1.726	5.673	0.495
Novel Group (n = 280)	7.384	0.323	5.501	0.690	3.105	0.484	5.30*	1.043	4.194	1.048	5.221	0.852

* ***p*** **<0.05, significant difference between RG and NG on affective assessment.**

#### Profile of Mood States (POMS)

The subjective questionnaire called Profile of Mood States (POMS) was selected to measure psychological status of Mars500 crew. This instrument has also been used in some Antarctic explorations as well as in general population studies due to its high test-retest and internal consistency reliability [26]. All crewmembers were required to finish this 5-point scale self-report questionnaire (‘1’ = lowest level, ‘5’ = highest level) for each testing. Mood data on 6 factors were obtained from POMS: tension-anxiety (T), depression-dejection (D), anger-hostility (A), fatigue-inertia (F), confusion-bewilderment (C), and vigor-activity (V). A Total Mood Disturbance (TMD) score was derived by summing the negative scores of five subscales (T, D, A, F, and C) and subtracting the unique positive score of V.

#### Plasma hormone sampling

To investigate the changes in plasma hormone levels, 7 mL of peripheral whole blood cells were extracted from each crewmember at approximately 7:00–8:00 in the morning, before breakfast. Two crewmembers with medical training took charge of blood sampling. After extraction, whole blood cells were immediately treated with EDTA as an anticoagulant, transferred outside of the module via an air-lock and centrifuged (at 3000 rpm for 15 min at 4°C) to collect the plasma. Of the obtained plasma, only 0.5 mL was used for the detection of cortisol, 5-hydroxytryptamine, dopamine and norepinephrine concentrations. The enzyme-linked immunosorbent assay (ELISA) kits (R&B, USA) were used according to the manufacturer's instructions. For assaying each parameter, each of the six samples was tested only once during the ELISA experiment process due to the limited amount of plasma available for this study.

### Protocols

All crewmembers learned the content and procedure of this study before isolation. During the confinement, the crew followed the working schedule to carry out 8 timed tests, which was sent into the module via specific local network ahead of one week. One crewmember was assigned to take charge of this study throughout the confinement. This duty included preparing the test device, reminding others to test on time, and collecting data and transferring them outside. On each testing day, a specific researcher was waiting for the data from the module in IBMP.

POMS and IAPS were manipulated on a 14-in screen Lenovo laptop (1280*800 pixels) with a mouse, and the presentation and recording were managed with Matlab 7.0 for WindowsXP. Every crewmember had a unique ID and password to run POMS and IAPS. They were asked to undertake POMS prior to IAPS. At the beginning of each test, the instructions were provided in three languages (Russian, English, and Chinese). After selecting the language version and reading the instructions, the crewmembers began testing. In POMS, there were 65 questions for the 5-point scale self-evaluation. In IAPS, the exposure time of each picture was 6 s and the assessing time was 16 s, with a 400-ms time interval before the next trial. The total time for POMS and IAPS was approximately 35 min. After operation, the data were saved and exported into a zip file named with the ID of the operator and the testing date (e.g., 5001_20100605.zip).

Caffeine and sport were avoided 2 hours before testing. All crewmembers were required to complete POMS and IAPS continuously in quiet surroundings. The six crewmembers took turns operating POMS and IAPS on the same laptop. Finally, the crewmember in charge of this study collected all zip files of each testing session and transferred them outside via the local network.

The specific days of blood sampling were close to the days of POMS and IAPS data acquisition (table 2). After collection and pretreatment, the blood samples were sent out immediately via a special air-lock.

**Table 2:** Mission days of tests and special events over the course of confinement.

	Mission Days(d)		
	POMS&IAPS	Blood Sampling	Special Events
Pre	-	−7	
Quarter 1	64	60	54 (Start of communication delay)
	119	120	
Quarter 2	166	168	181(Power emergency simulation)
			244 (Mars Lander hatch opening)
	241	249	257–265 (Egresses on Mars surface)
			272 (Hatch closure, Lander undocking)
Quarter 3	305	300	320 (Communication error simulation)
	366	360	351(Max communication delay)
Quarter 4	421	418	470 (End of communication delay)
	512	510	
Post	-	+7	Debriefing of the crew

### Statistical analysis

The changes of affective assessment on IAPS were analyzed with ANOVA using two within-factors: ‘pictures’ (unpleasant/pleasant/neutral) and ‘mission days’ (8 levels without pre and post, table 2).

The collected mood scores with non-normal or skewed distributions (T, D, A and F) were first square-root transformed. Then, with normally distributed C and V, the data of POMS were allowed for standard parametric analysis. Repeated-measures ANOVA was used for comparisons of changes in mood state over isolation.

Percentage changes in blood biochemical data between baseline (−7 d) and each day-point were calculated to show the varying plasma hormone levels. Repeated-measures ANOVA was used for comparisons of changes in plasma hormone levels over the course of isolation.

The preserved Greenhouse-Geisser method was chose for Mauchly's Test of Sphericity. Bivariate comparisons were conducted using Spearman correlation coefficients to determine the association between the affective assessment and the changes in the psychological and biochemical results. Bonferroni's test was used for post-hoc analysis. The significance level was set to *p*<0.05, and values are shown as the mean ± SD.

## Results

### Changes in IAPS

#### Positive bias on valence rating

**Figure 1:**
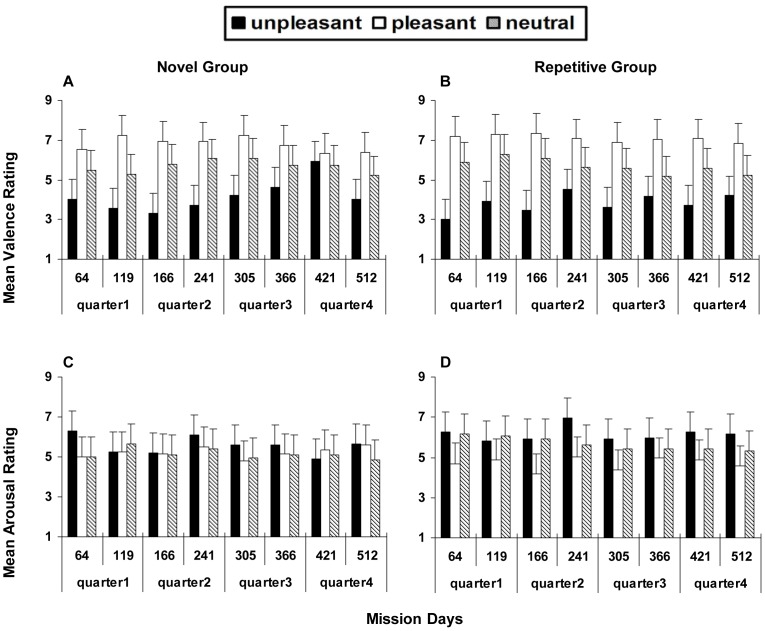
Mean valence and arousal ratings of affective stimuli from the NG and RG. (A) Valence rating of NG. (B) Valence rating of RG. (C) Arousal rating of NG. (D) Arousal rating of RG.

In NG (figure 1.A), ANOVA on the mean valence rating showed a significant main effect for ‘pictures’ (F _2, 10_ = 14.507, *p*<0.01). The rating for pleasant pictures was significant higher than that for unpleasant pictures (*p* = 0.026) and neutral pictures (*p*<0.01). The effect of ‘mission days’ showed no significance. The interaction of ‘mission days * pictures’ had a significant effect (F _14, 70_ = 4.24, *p* = 0.017). Although it seemed that the unpleasant pictures rating on day 421 was the highest over time, post-hoc tests exhibited no significance. Post-hoc comparisons showed a significant difference between the unpleasant pictures rating and pleasant pictures rating over time except on day 366 (*p* = 0.06) and day 421 (*p* = 0.052). The shrinking gap between the assessment of unpleasant and pleasant pictures revealed a bias in which the crew tended to assign positive ratings to the unpleasant pictures at those times. For the pleasant and neutral pictures, this positive bias did not appear.

**Figure 2:**
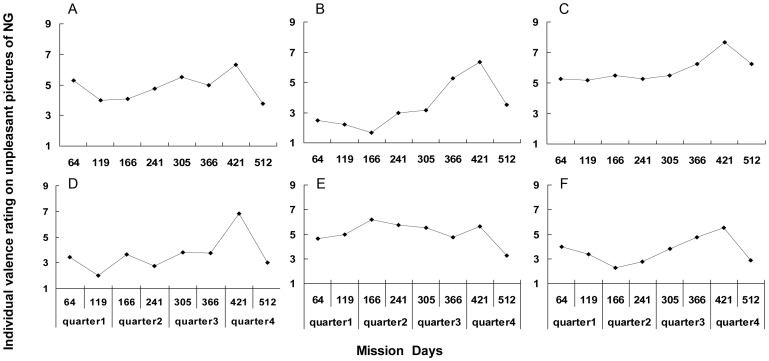
Individual valence ratings of unpleasant pictures from the NG. (**A**)–(**F**), **valence ratings from each crewmember.**

Regarding the impact of individual difference on small n = 6, we analyzed each crewmember's data separately (figure 2). We found that this specific change of unpleasant pictures rating in NG was obvious among all crewmembers except ‘E’ (figure 2.E). Therefore, this positive bias on valence rating was not due to individual difference.

In RG, ANOVA on mean valence rating only showed a significant main effect only for ‘pictures’ (F 2, 10 = 15.331, *p* = 0.01) (figure 1.B). The unpleasant pictures rating was significantly lower than the ratings of neutral (*p* = 0.011) and pleasant pictures (*p* = 0.013) in RG. There were no significant effects for ‘mission days’ or ‘mission days * pictures’.

#### Stable arousal rating

For both RG and NG, ANOVA conducted on arousal rating showed no significant change (figure 1.C and D). Compared with the valence rating, the changes of arousal rating were stable.

### Changes in POMS

**Figure 3:**
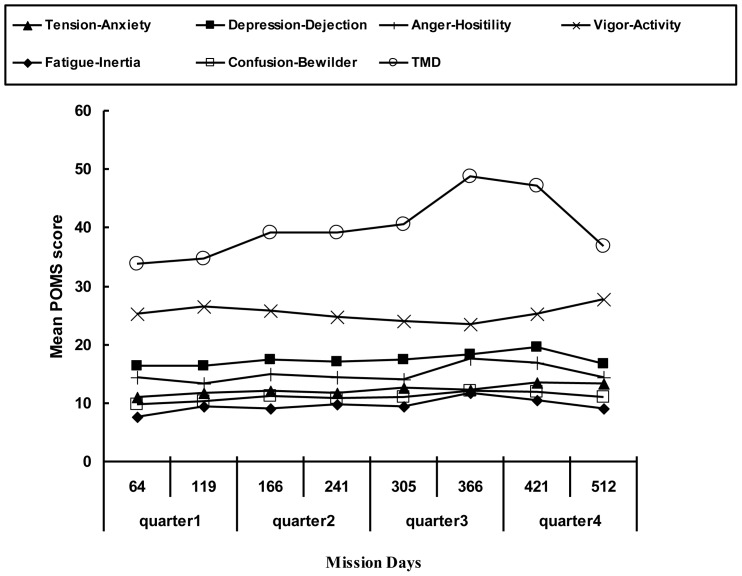
Mean POMS scores collected over 520 d confinement. The score of vigor-activity fluctuated significantly over the course of confinement (*p*<0.05).

The mean POMS score fluctuated during isolation (figure 3). The score of vigor-activity changed significantly over time (F _7, 35_ = 3.515, *p* = 0.041); however, the pos-hoc comparisons showed no significant difference. Though TMD seemed to fluctuate in a stage model, the statistical result was not significant. The scores for TMD and vigor were temporally related, with each reaching their extreme value on day 366.

### Changes in plasma hormones

Paired-*t* comparisons between pre (−7 d) and post (+7 d) plasma samplings showed subtle decreases in cortisol and DA (table 3). A significant rise was evident for both 5-HT (*p* = 0.009) and NE (*p* = 0.002).

**Table 3:** The mean changes in plasma hormones.

Plasma Hormones		Mission Days									
		−7 d	60 d	120 d	168 d	249 d	300 d	360 d	418 d	510 d	+7 d
Cortisol (mkg/dl)	Mean	18.39	13.19	17.18	17.16	18.81	14.36	21.29	15.13	23.75	15.84
	SD	4.35	2.65	4.08	3.53	5.39	4.97	5.59	7.96	3.91	6.59
5-HT (ng/ml)	Mean	239.03**	261.44	213.45	200.98	426.64	379.10	385.07	311.61	297.67	324.36
	SD	59.31	35.37	32.61	39.66	51.30	59.22	28.64	59.34	52.13	46.91
DA (ug/ml)	Mean	1.16	1.34	1.41	1.41	1.07	1.16	1.07	1.04	1.04	1.08
	SD	0.23	0.22	0.12	0.48	0.04	0.21	0.04	0.02	0.01	0.09
NE (pg/ml)	Mean	412.82**	458.58	528.32	505.76	781.61	873.31	745.29	650.16	624.31	623.11
	SD	83.22	82.77	113.21	97.21	100.43	153.59	73.91	107.49	118.98	34.49

**Paired-** ***t*** **comparison between pre (−7 d) and post (+7 d) blood samplings,**

* ***p*** **<0.05,**

** ***p*** **<0.01.**

**Figure 4:**
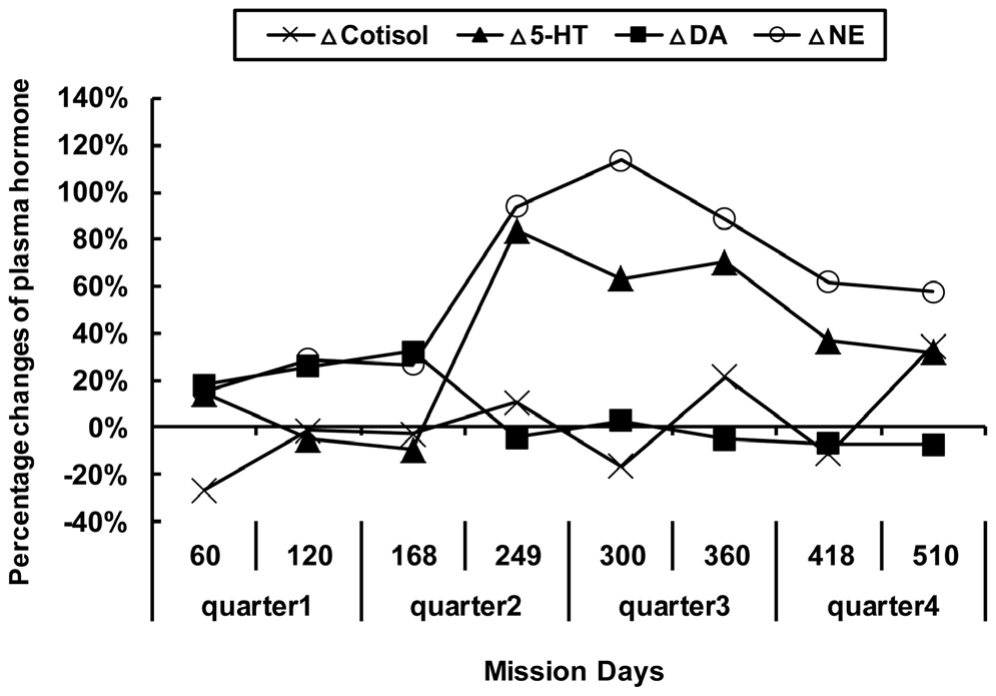
Percentage changes of plasma hormone level compared with baseline (−7 d) over 520 d confinement. Δ5-HT, ΔCortisol and ΔNE fluctuated significantly over the course of confinement (*p*<0.05).

As shown in figure 4, the level of NE rose significantly between day 168 and day 300. After that, it began to decline, but it remained higher than the first 3 day-point collections (F _7, 35_ = 9.415, *p*<0.01). The level of 5-HT changed in much the same pattern as NE (F _7, 28_ = 16.377, *p* = 0.001). As shown in table 2, it could be seen that the Mars landing period (from day 244 to day 272) was close to the time of this rise in 5-HT and NE. The level of cortisol exhibited an increasing tendency with obvious fluctuation (F _7, 35_ = 4.724, *p* = 0.016), and it reached its maximum value on day 510. The level of DA showed a quasi-significant decline on day 249 (F _7, 35_ = 4.006, *p* = 0.067), and it then remained relatively stable until the end of the study.

### The association between affective assessment and mood and plasma hormone levels

The negative mood scores for depression, anger and TMD had significant positive correlations with Δ5-HT and ΔDA (table 4), which were negatively associated with vigor (*p*<0.05). ΔNE was positively correlated with vigor and TMD (*p*<0.05).

**Table 4:** Spearman correlations between percentage changes of hormone levels and mood score.

				Mood score			
Percentage changes of hormone level	Tension -Anxiety (T)	Depression -Dejection (D)	Anger -Hostility (A)	Vigor -Activity (V)	Fatigue -Inertia (F)	Confusion -Bewilder (C)	Total Mood Disturbance (TMD)
ΔCortisol	0.109	−0.099	0.131	0.117	0.295*	0.345*	−0.077
Δ5HT	0.124	0.384**	0.328*	−0.558**	0.226	−0.035	0.559**
ΔDA	0.259	0.592**	0.360*	−0.608**	0.213	−0.064	0.549**
ΔNE	0.219	0.230	−0.303*	0.330*	0.229	−0.139	0.407**

* ***p*** **<0.05,**

** ***p*** **<0.01, significant Spearman correlating coefficients.**

The valence rating for novel unpleasant stimuli was significantly correlated with depression, vigor, fatigue, TMD, ΔNE and Δ5-HT (table 5). The associations between affective assessment and changes in mood and hormone levels existed and reflected the stability of the behavioral performance and the psycho-physiological status.

**Table 5:** Spearman correlations between valence rating on affective stimuli and mood score and percentages of hormone level.

Valence rating		Mood score							Percentage changes of hormone level			
		T	D	A	V	F	C	TMD	ΔCortisol	Δ5HT	ΔDA	ΔNE
NG	unpleasant	0.026	0.315*	0.182	−0.456**	0.287*	0.157	0.473**	−0.212	0.328*	0.146	0.270*
	pleasant	−0.381**	−0.652**	−0.365*	0.643**	−0.412**	−0.292*	−0.670**	−0.044	−0.387**	−0.363*	−0.140
	neutral	−0.442**	−0.507**	−0.296*	0.454**	−0.364*	−0.128	−0.442**	−0.146	−0.051	−0.424**	0.058
RG	unpleasant	0.203	0.551**	0.166	−0.522**	0.398**	0.286*	0.599**	−0.197	0.526**	0.437**	0.378**
	pleasant	−0.518**	−0.746**	−0.378**	0.707**	−0.400**	−0.333*	−0.734**	0.080	−0.455**	−0.485**	−0.325*
	neutral	−0.373**	−0.212	−0.295*	0.207	−0.207	−0.366*	−0.268	−0.373**	−0.203	0.108	−0.218

* ***p*** **<0.05,**

** ***p*** **<0.01, significant Spearman correlating coefficients.**

## Discussion

One salient finding of this study was the positive or neutral bias on valence rating of novel unpleasant stimuli over the prolonged confinement. Although IAPS has never been used in studies of ICEs before, we found several pieces of evidence about affective assessment under some relevant stressors. For instance, Weinberg [27] found a similar emotional response in patients with Generalized Anxiety Disorder (GAD). Del Seppia [28] reported that the impact of geomagnetic stimulation might enhance autonomic responses to emotional stimuli. Tempesta [29] found that one-night sleep deprivation influenced the balance rating of neutral stimuli on a negative bias. Yoo [30] found different results that were still consistent with the adverse effects of sleep deprivation, and increased negative evaluations of neutral stimuli.

However, none of these studies concerned the accumulating impact of a long duration of stress, which might be the critical factor causing the positive bias on valence rating in the present study. It has been proved that human behavior can reflect the current mental state [31], [32]. In our opinion, the positive bias evaluation on negative stimuli was influenced by the aggravating psychological stress over time, which was consistent with the fluctuation of mood and hormone levels. With the influence of time, it was internal reluctant for the crewmembers to face negative stimuli which might make their personal state further worse. To avoid serious mental feelings and issues, the crew tended to assign positive ratings to these negative stimuli. On the other hand, the higher the valence rating on the unpleasant pictures, the worse the psychological status of the crew might be.

Humans have evolved to think adaptively rather than logically in many contexts, depending on the relevant factors. From the perspective of evolutionary psychology, this has been summarized as adaptive defense in reference to cognitive distortions [33]. For example, negative emotional stimuli and responses are associated with the defensive system that mediates withdrawal, escape from harm and defensive aggression behaviors. Driven by the defensive system, unpleasant emotions, such as anxiety, depression and fear, induce different reflexive automatic and somatic outputs [34], [35], [36], [37]. In the current study, when the psychological status of the crew became worse with time, they expressed withdrawal from the negative stimuli with a positive rating bias.

In addition to time, there were other factors inside which might impact the crew over confinement: the special events in table 2, the repetitive boring psychological questionnaires, and the heavy workload fitness tests, among others. However, in the current study, we think it is difficult to define their contribution to our principal finding. First, the effects of most factors on each crewmember were acute and inconsistent, thus differing from the chronic mild effect of time. For example, after filling out monotonous questionnaires for more than an hour, some crewmembers felt much upset and low every time, while some felt normal. Further, the negative feeling which haunted them varied in duration. Some factors that might have the possibility to cause positive response, such as the Mars landing activities, also showed varying effects on the six crewmembers. Second, other impacting factors were not consistent with the collected data which we used to conclude our previous finding. Some of them were even random and inescapable, for instance, the power emergency simulation. Therefore, we believe that time was the main factor contributing to the principal finding in this study. Whether this phenomenon would happen under short- or medium-term confinement, and whether it would be caused by other factors, must be examined in the future.

Researchers employ periods of inescapable stress to explore the putative psychopathology of affective responses and disorders. Although the neural mechanisms remain unclear, the neuroendocrine system seems to play an important role. For instance, hypothalamic pituitary-adrenal (HPA) axis abnormalities are well established in major depression [38], [39], [40]. The function of the serotonin (5-HT) system is important to mediate stress although the evidence is less consistent [21], [41], [42]. However, the increased psycho-physiological resistance to chronic stressors such as depression and anxiety is most likely related to the increased release of 5-HT [43], [44], [45]. In our study, we found that the assessment of novel negative pictures was correlated with the changes in mood score and 5-HT and NE levels. Combining the psychological patterns with biochemical data, we deduced that there was a stage-changing adaptation for the Mar500 crew over 520 days of confinement: primarily stable at the beginning (quarter 1); then obvious changes appeared, such as the elevation of 5-HT and the decline of vigor, after the midpoint or near the end of quarter 2; the changing climax emerged in quarter 3 or near the beginning of quarter 4; and, finally, the fluctuating amplitude shrank towards the initial value from quarter 1. The adaptive pattern of the Mars500 crew is similar to the third-quarter phenomenon.

On +7 d after isolation, a detailed interview recording with both hand and video was made for each crewmember. The log, written by one crewmember, who was also the first author of this manuscript, provided extra reference. From the crew's viewpoints, the enthusiasm and high motivation dominated primarily, which was helpful in conquering anxiety and maladaptation to the new environment, daily tasks and teammates. This ‘happy’ adaptive duration lasted for 2–3 months, and then negative feelings (depression, inertia, monotony, etc.) became dominant. This situation generally ameliorated as the Mars landing approached. During the landing period, the crew was exposed to some novel and inescapable stressors, especially for the three landing members. These new stressors included supplying materials, head-down bed rest, anti-G suit, extravehicular activity (EVA) simulation, and driving robot detector, etc. They promoted the crew's enthusiasm and energy rather than impairing their interest and performance. Accordingly, the rise in 5-HT and NE was temporally correlated with mid-mission activities, which was related to stress coping. This change was similar to those of some previous studies [43], [45]. After the Mars landing, there were no further novel stimuli. Monotony, depression, repeated scientific experiments and dehydrated food became routine. Once having the thought that almost half the time remained, the crewmembers found it difficult to calm down and sleep well. Except for the specific changes of emotional response in our study, some evident deterioration of mood, sleep, physical activity and neuromuscular performance was found at that moment [9], [10], [11]. Additionally, the crew's socialization time outside of work became less. Most of the crewmembers lost their interest in communicating. Instead, they locked themselves in their private capsules. Even new mails from outside were difficult to excite them. This tough period lasted for approximately 4–5 months. When the crew realized that the remaining time was approximately 2–3 months, the psychological status began to improve. For instance, the frequency of team communication and entertainment in their spare time increased. However, the sensitivity and intensity of crew's response to internal and external stimuli also increased. For example, impatience at new requests from outside increased, and disagreement within teammates and conflict with outside operators occurred more often [46], [47]. This state lasted until the exit.

This stage-changing model in Mars500 reflected by psychological and biochemical data was not completely identical with the crew's description. The mid-mission activities might make a major contribution to the difference, which was emphasized by the Mars500 crew and also reflected on hormone levels during the stress-coping periods. If we overlooked the period of the Mars landing, the adaptive pattern with stages was generally accorded with the crew's descriptions. In addition, novel tasks during the Mars landing period seemed helpful to activate motivation and attenuate the negative psychological effects, especially depression and monotony. This would be applicable to arranging the work scheme for astronauts on a long-term spaceflight.

The limitations of this study include the non-controlled design and the small sample size, which also occurs in real space missions and polar expeditions. Non-coincidence between testing days of IAPS & POMS and blood sampling days might influence the results, but it was impossible to arrange them on absolutely identical schedules in the Mars500 project. However, the correlation between biochemical and psychological data was significant. We identified that the Mars500 crew exhibited a stage-changing pattern of psychological adaptation over confinement, which is similar to the third-quarter phenomenon. Additionally, based on the debriefing of the Mars500 crew and our data analysis, we believed both the third and last quarter needed to be paid more attention. Because mental issues, which are essential not only to the crew's health but also to the success of the whole mission, would most likely occur and effect during that period.

## Conclusions

In this study, the data of IAPS indicated a positive bias on valence rating over isolation. It would be associated with the defensive system, which was probably activated by the aggravating psychological stress with time. The Mars500 crew exhibited a stage-changing pattern of psychological adaptation during 520 days of confinement, which is similar to the third-quarter phenomenon.
